# Interplay Among Synaptic Glutamate Release and Excitotoxicity: Neuronal Damage and Graphene-Based Materials Related Protection

**DOI:** 10.3390/life15111776

**Published:** 2025-11-19

**Authors:** Giada Cellot, Laura Ballerini

**Affiliations:** 1 Neuroscience Area, International School for Advanced Studies (SISSA), Via Bonomea 265, 34136 Trieste, Italy; 2 Department of Life Sciences, University of Trieste, Via Giorgieri 5, 34127 Trieste, Italy

**Keywords:** excitotoxicity, glutamate, nanomaterials, graphene, synapse, glutamatergic receptors, calcium dysregulation, drug delivery, secondary damage

## Abstract

Glutamate-related excitotoxicity represents a fundamental pathological process underlying both acute and chronic disorders of the central nervous system. Excessive stimulation of ionotropic and metabotropic glutamate receptors induces ionic dysregulation, mitochondrial dysfunction, and oxidative stress, which can activate necrotic and apoptotic pathways, processes further amplified by defective glutamate clearance and astrocytic impairment. These mechanisms are recognized as key contributors to neuronal damage in ischemic stroke, Alzheimer’s disease, Parkinson’s disease, and Huntington’s disease, identifying excitotoxicity as a convergent hallmark of neurodegeneration. Despite considerable progress in elucidating its molecular mechanisms, clinical translation of excitotoxicity-targeted interventions remains limited, largely due to the difficulty of selectively attenuating pathological glutamatergic activity while preserving physiological neurotransmission. Recent advances in nanotechnology, particularly the development of graphene-based materials (GBMs), have offered innovative approaches for neuroprotection. Owing to their unique physicochemical properties and compatibility with neural tissue, GBMs have been investigated as platforms for neural interfacing, regenerative scaffolds, drug delivery platforms, and direct modulators of glutamatergic transmission. In particular, small graphene oxide nanosheets exhibit the capacity to downregulate glutamate release and confer anti-inflammatory and neuroprotective effects. These findings suggest that GBMs may represent a promising class of neuromodulatory tools for mitigating excitotoxic injury, warranting further preclinical and translational investigations.

## 1. Glutamate-Induced Excitotoxicity

At physiological concentrations (1–5 µM in the extracellular space), glutamate is the major excitatory neurotransmitter in the mammalian central nervous system (CNS) and a key mediator of intercellular communication, plasticity, growth, and differentiation [1]. Due to this crucial role in fundamental CNS processes, glutamate release, uptake, metabolism, and signaling are physiologically tightly regulated [2]; however, different pathological states, from traumatic insult to neuro-diseases, may alter glutamate control mechanisms and result in increased glutamate concentration in the CNS extracellular space, a phenomenon that can trigger neuronal dysfunction and neurodegeneration [3]. Experimentally, acute excitotoxic effects are observed when extracellular glutamate concentrations exceed roughly 100 µM within minutes to hours, while chronic exposure to moderately elevated levels of 10–50 µM over extended periods contribute to progressive neurodegeneration [4].

The potential toxicity of glutamate was first described in the mouse retina, where the inner layers degenerated after subcutaneous injections of glutamate [5]. These results were confirmed in the whole brain following intracranial injection of glutamate in both mouse and primate models [6, 7]. These authors coined the term “glutamate excitotoxicity”, hypothesizing that glutamate receptors’ overactivation are a primary cause of neuronal loss [8]. In later studies, similar neuronal degeneration was observed following cerebral anoxia [9] with the reported involvement of glutamate receptors’ activation shown by the reduced susceptibility to anoxic insult in in vitro hippocampus when treated with the non-specific postsynaptic excitatory amino acid inhibitor gamma-D-glutamyglycine [10]. Indeed, glutamate receptors-mediated excitotoxicity is considered a major mechanism shaping the spatial–temporal profile of secondary CNS damage after acute events [11].

Although initially the research in glutamate excitotoxicity focused on acute CNS damages, such as ischemia or traumatic brain injury, later on the alteration in glutamate homeostasis was definitely linked to the progression of secondary damage and of chronic neurodegenerative disorders, such as amyotrophic lateral sclerosis (ALS), Alzheimer’s and Parkinson’s diseases (AD and PD, respectively) [12].

## 2. Glutamate Receptors and Their Role in Excitotoxicity

Glutamate acts through two main classes of receptors: ligand-gated ion channels, named ionotropic glutamatergic receptors, and G-protein coupled receptors, i.e., metabotropic glutamatergic receptors (mGluRs), both types being expressed by neuronal and glial cells.

### 2.1. Ionotropic Glutamatergic Receptors: An Overview

Within ionotropic glutamatergic receptors, the α-amino-3-hydroxy-5-methyl-4-isoxazolepropionic acid receptors (AMPARs) are tetramers composed by different subunit (GluA1–A4) combinations, providing diverse receptor properties, such as trafficking, cellular distribution, post-translational modifications and biophysical features [13]. AMPARs activation mediates fast postsynaptic responses to glutamate released from the presynaptic site, leading to an inward current predominantly mediated by mixed cations (sodium and potassium) flux across the membrane [14]. The majority of AMPARs contain GluA2 subunits, which are responsible for the low calcium permeability of the receptors [15], although depending on their editing status [16], while GluA2-lacking receptors are typically calcium-permeable [17], and recent evidence shows that they play a main role in non-Hebbian form of synaptic plasticity [18]. Of interest, the activation of calcium-permeable AMPARs, either due to subunit expression or editing [16], is also involved in neurotoxicity, during acute CNS insults, such as ischemia [19].

Kainate receptors (KARs) are heterotetramers of GluK1-5, whose combinatorial effects lead to different receptor conductance. Similarly to AMPARs, KARs present fast kinetics and a higher permeability to sodium rather than to calcium ions [20], although their calcium permeability can be affected by Q/R editing of the GluK1 and GluK2 subunits [21]. In neurons, while AMPARs are localized mostly in the postsynaptic membrane, kainate receptors can be localized in both the presynaptic [22] and postsynaptic sites [23]. In excitotoxicity, both AMPARs and KARs contribute to neuronal damage, triggering membrane depolarization, which, in turn, is responsible for enhanced calcium influx into neurons [24]. In parallel, when expressed and activated, calcium-permeable AMPARs and KARs can directly mediate the rise in intracellular calcium concentration, igniting the mechanisms leading to neurotoxicity [16, 21].

Among ionotropic glutamatergic receptors, *N*-methyl-d-aspartate receptors (NMDARs) have attracted interest for the plethora of physiological and pathological processes in which they are involved [25]. Structurally, the receptor is composed of three different subunits, GluN1-3 and, usually in the form of a heterotetramer, made up of two GluN1 subunits and two GluN2 subunits [3]. Differently from the other two ionotropic glutamatergic receptors, NMDARs exhibit slow kinetics and a complex gating mechanism, where receptor activation requires the simultaneous binding of glycine and glutamate [26] and contextual cell membrane depolarization, to remove the voltage-gated Mg^2+^ block of the channel pore [27]. GluN1 subunits are mainly responsible for these peculiar properties, although subtype-specific differences of Mg^2+^ block and other heterogeneous biophysical properties are determined by the GluN2 subunits [28, 29].

In physiological conditions, the depolarization necessary to activate the receptor can be triggered by the co-activation of AMPARs and KARs. Once opened, NMDARs are mainly permeable to sodium and calcium ions, a process involved in the induction of Hebbian synaptic plasticity [30]. However, when the activation of these receptors is temporally prolonged, the resulting intracellular calcium overload can induce cell dysregulations, leading to neurotoxicity, whose mechanisms are described below.

A comprehensive review regarding ionotropic glutamate receptor pharmacology can be found elsewhere [31].

### 2.2. Metabotropic Glutamatergic Receptors: An Overview

mGluRs are single-peptide seven-transmembrane-spanning proteins linked to intracellular G-proteins [32]. Based on preliminary observations, mGluRs were thought to act exclusively through the activation of G-proteins [33], but more recently mGluRs were reported to exert their modulatory activity via G-protein-independent signaling cascades [34]. mGluRs can cooperate with ionotropic glutamatergic receptors with variable mechanisms, such as direct interactions, interaction via scaffold proteins, shared downstream targets, modulation of new glutamate receptors’ transcription and of old receptors’ internalization [35]. At least eight types of mGluRs have been cloned (mGluR1-8) and classified, based on the sequence homology and their intracellular effects, in three groups: I, II, and III mGluRs [36].

Group I mGluRs includes mGluR1s and mGluR5s, where glutamate binding activates phospholipase C (PLC) that, among its downstream effects, is responsible for inositol triphosphate (IP3) production and subsequent intracellular calcium mobilization [37, 38]. Dysfunctional signaling via group I mGluRs is thought to lead to defective internalization of AMPARs, which may also influence the permeability of the cellular membrane to calcium [39]. In excitotoxic conditions, the group I mGluRs can potentiate NMDARs-mediated Ca^2+^ influx at the postsynaptic site [40].

Group II mGluRs include mGluR2s and mGluR3s. These receptors are expressed at both the presynaptic and the postsynaptic site [41] and are responsible for the inhibition of voltage-gated calcium channels by decreasing the adenylyl cyclase (cAMP) formation [42, 43]. Through this mechanism, group II mGluRs tune neurotransmitter release and neurotransmission.

The activation of group III mGluRs, which include mGluR4s, mGluR6s, mGluR7s, and mGluR8s, is also associated with a reduction in cAMP signaling, with downstream inhibition of voltage-gated calcium channels [43]. This group of mGluRs are also expressed at both the presynaptic and postsynaptic compartments [44]. They can modulate neurotransmission by regulating calcium channels at presynaptic terminals, working as auto-receptors [45].

In sum, mGluRs can have a dual and nuanced role in excitotoxicity. Whereas group I mGluRs can exacerbate damage, by increasing intracellular calcium via the PLC–IP3 pathway, enhancing NMDA-receptor-mediated calcium entry and promoting oxidative stress, group II and group III mGluRs can counteract it by acting mainly at presynaptic level to suppress glutamate release.

## 3. Highlights of the Excitotoxicity Mechanisms

**Figure 1:**
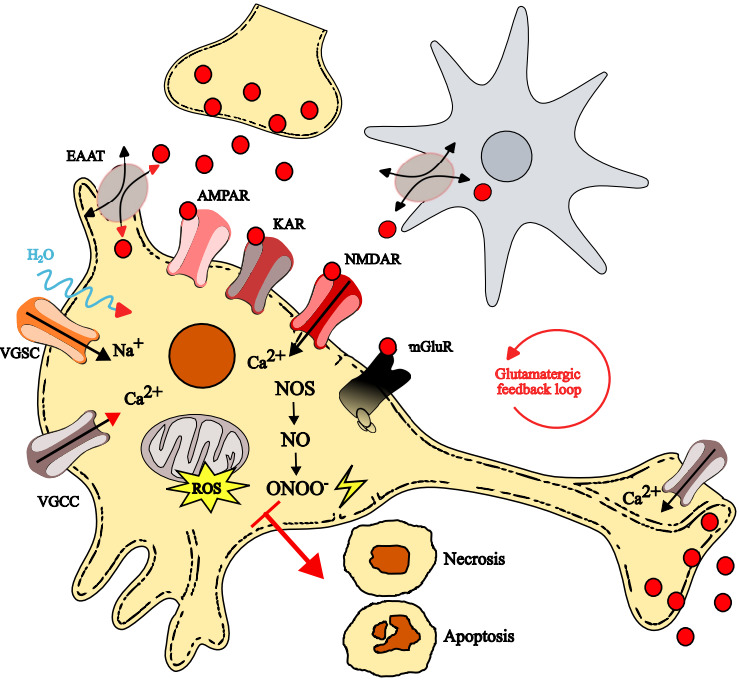
Mechanisms of excitotoxicity leading to neuronal damage. Excessive glutamate (red dots), released in the extracellular space, activates ionotropic (AMPARs, KARs, and NMDARs) and metabotropic (mGluRs) glutamatergic receptors. AMPARs and KARs opening drives cell membrane depolarization, which in turn activates voltage-gated sodium (VGSCs) and calcium (VGCCs) channels, leading to ionic imbalance, water inflows and cell swelling. Depolarization also relieves the magnesium block of NMDARs, enhancing calcium entry. Elevation of intracellular calcium concentration activates nitric oxide synthase (NOS), which produces nitric oxide (NO), which, together with calcium overload, disrupts mitochondrial function. Such mitochondrial dysfunction generates reactive oxygen species (ROS) and peroxynitrite (ONOO^−^), ultimately triggering necrotic or apoptotic cell death. Energy failure impairs EATTs activity of glutamate re-uptake in both glial (in gray) and neuronal (in yellow) cells, worsening the glutamatergic feedback loop.

During excitotoxicity, three interrelated factors contribute mostly to the cascade, leading to neuronal injury: glutamate exocytosis-driven processes, sodium influx- and calcium influx-dependent events. These pathways are not discrete but cooperate interactively, amplifying excitotoxic signaling, and thus investigating how these mechanisms contribute to glutamate-mediated cell death will provide not only insights into the molecular basis of CNS excitotoxic damage (Figure 1), but will also indicate potential targets for therapeutic intervention. The next paragraphs briefly describe the phenomena occurring during excitotoxicity.

### 3.1. Sodium-Driven Osmotic Dysregulation and Its Role in Neuronal Injury

Conspicuous glutamate released from the presynaptic site binds to AMPARs and KARs, resulting in postsynaptic depolarization, and neuronal activation of voltage-gated sodium channels, leading to sodium influx. Sustained depolarization also disrupts the cell’s osmotic homeostasis since the increased intracellular sodium concentrations [46] determine passive chloride ions cell entry in the attempt to preserve the electrochemical balance [47]. As a consequence, water inflows into the cell along the resulting osmotic gradient, with cellular swelling and progressive dilution of the cytoplasmic contents, ultimately compromising organelle integrity. This process can culminate in cell lysis and in an uncontrolled release of intracellular components into the extracellular space [48], including, once more, glutamate.

While sodium dysregulation contributes to neuronal injury—particularly if severe enough to cause lysis—it is not the leading process in excitotoxic cell death. In fact, the removal of extracellular sodium and chloride ions, while preventing osmotic swelling, does not avoid cell death [49], supporting the pivotal and non-redundant role of calcium-dependent processes in driving irreversible neurotoxicity during the excitotoxic cascade (see below).

### 3.2. Amplification of Neuronal Damage via the Glutamatergic Feedback Loop

During excitotoxicity, extracellular glutamate concentration rises markedly due to three mechanisms: (1) passive release of cytosolic glutamate from lysed neurons, (2) impaired or reversed function of excitatory amino acid transporters (EAATs), usually committed to glutamate re-uptake after sustained depolarization; and (3) calcium-dependent exocytosis of glutamate-containing synaptic vesicles. Elevated extracellular glutamate exacerbates depolarization in already-compromised neurons and propagates excitotoxic signaling to neighboring cells. This positive feedback loop amplifies excitotoxic damage and facilitates the spreading of neurodegeneration within affected regions [50]. A comprehensive description of EAATs and their role in exacerbating excitotoxicity can be found elsewhere [51].

### 3.3. Calcium-Dependent Mechanisms Driving Excitotoxic Neuronal Death

Calcium influx through NMDARs is essential for glutamate-induced excitotoxicity [43]. Glutamate neurotoxicity may be partially blocked by antagonists of NMDARs [52]. Notably, calcium influx through NMDARs triggers neuronal mortality more efficiently, when compared to larger influxes via other calcium-permeable channels [53], such as voltage-gated calcium channels, calcium-permeable AMPARs, or via sodium/calcium exchangers (NCE) and proteins releasing calcium from intracellular stores. Elevated intracellular calcium concentrations induce cell death through multiple pathways, including activation of nitric oxide synthase (NOS), calcium-dependent proteases, and mitochondrial dysfunction [54, 55].

Neuronal nitric oxide synthase (nNOS) is closely associated with the NR2 subunit of NMDARs through the scaffolding protein postsynaptic density 95 (PSD95), creating a postsynaptic microenvironment that favors calcium influx activation of nNOS via calmodulin binding, leading to the production of neurotoxic levels of nitric oxide (NO) [56]. NO interacts with various intracellular targets, including superoxide radicals to form peroxynitrite (ONOO^−^), [57], a potent oxidant that induces protein nitration, lipid peroxidation, and DNA damage, resulting in cell death [58, 59].

More recently, NO has been implicated in an additional neurotoxic pathway responsible of an apoptotic-like form of cell death, which involves glyceraldehyde-3-phosphate dehydrogenase (GAPDH) and the E3 ubiquitin ligase Siah1 [60]. Under physiological conditions, GAPDH works as a key enzyme in glycolysis; however, under conditions of elevated NO, GAPDH undergoes S-nitrosylation [61], and binds Siah1-forming complexes translocating to the nucleus. There, it promotes p300/CBP-mediated acetylation of nuclear proteins such as p53, triggering apoptotic features, including pyknotic nuclei and cell death [60]. In parallel, this interaction may act as a metabolic switch. By sequestering GAPDH from the cytosol, NO can interfere with glycolytic flux, potentially resulting in energy failure and necrotic cell death [62]. Thus, the NO–GAPDH axis represents a dual-threat mechanism, capable of promoting both apoptotic and necrotic pathways depending on the cellular context.

Evidence indicates that during excitotoxicity, much of the intracellular calcium is sequestered by mitochondria [63]. Such mitochondrial calcium overload promotes metabolic acidosis and the generation of reactive oxygen species (ROS), exacerbating mitochondrial dysfunction [64]. Antioxidant treatments, including radical scavengers, confer neuroprotection against glutamate excitotoxicity [65]. ROS generated in mitochondria can interact to form highly reactive species, for example, peroxynitrite, which inhibits mitochondrial electron transport chain complexes I and II [66] and disrupts cytochrome c function [67]. These effects facilitate caspase activation, promoting once more apoptotic cell death [3].

Transient cytoplasmic calcium elevations also activate calpains, which are calcium-sensitive cysteine proteases. μ-calpain’s proteolytic activity is needed to cleave and release apoptosis-inducing factor (AIF) from mitochondria [68]. AIF release triggers chromatin condensation, DNA fragmentation, and subsequent cell death [69]. Another pathway involves the nuclear DNA repair enzyme poly(ADP-ribose) polymerase-1 (PARP-1), which, when overactivated, generates poly(ADP-ribose) (PAR) polymers essential for AIF release [70]. Overactivation of PARP-1 appears to require NO formation, linking NR2 subunit of NMDAR, calcium permeation and nNOS upregulation to multiple excitotoxicity pathways [71]. This suggests a possible interplay between the calpain/AIF and PARP-1/AIF pathways in mediating excitotoxic cell death [72].

A detailed description of necrotic/apoptotic pathways activated by excitotoxicity can be found elsewhere [73].

### 3.4. Astrocytic Dysfunction and Its Contribution to Glutamate Excitotoxicity

Astrocytes are at the core of glutamate homeostasis; these glial cells tightly regulate the neurotransmitter concentration at the synaptic cleft via high-affinity transporters [74]. However, under pathological conditions, astrocytic function can become severely compromised, up to the stage of contributing significantly to the excitotoxic cascade [75]. Glutamate uptake by astrocytic co-transporters may be impaired by ATP depletion, leading to the reversed operation of EAATs, promoting glutamate efflux and exacerbating excitotoxicity. Additionally, in astrocyte, the ATP-dependent conversion of glutamate to glutamine via glutamine synthetase may be disrupted, with the progressive buildup of intracellular glutamate. This not only hampers glutamate uptake but also increases the likelihood of reversed transport, further amplifying extracellular glutamate levels [76]. Astrocytes also express both ionotropic and metabotropic glutamate receptors, and under pathological conditions, the expression of NMDARs is upregulated [77], increasing their vulnerability to glutamate-mediated toxicity. The commitment of astrocytes to glutamate excitotoxicity has been reviewed elsewhere [78]; briefly, astrocytes are actively involved in neurodegenerative disorders by mediating relevant and specific processes, as distinct as neuroinflammation, oxidative stress, extracellular vesicles contribution, or protein misfolding [79]. In neurodegenerative diseases where glutamate excitotoxicity is the dominant pathological feature, a specific contribution by astrocytes has been proposed, for example, in ALS [80]. There are multiple molecular mechanisms involved in astrocytes’ participation in the increased extracellular glutamate levels and in excessive glutamate toxicity to astrocytes itself; indeed, recently, astrocytes have been indicated as both “producers” and “targets” of excitotoxicity [80]. Excessive glutamate can originate directly (from neuronal and astrocyte release) and indirectly (reduced uptake), thus there are various pathways by which astrocytes in pathological conditions become producers of glutamate excitotoxicity. Similarly, by various mechanisms, they became targets of their released glutamate, leading to a vicious circle, where reactive astrocytes have a detrimental effect on neighboring neuronal and glial cells [81]. In addition, it has been reported that astrocyte swelling and impairment can be activity-dependent and mediated by pH-regulating mechanisms [82].

## 4. Excitotoxicity Role in Secondary Damage Development: The Case of Ischemic Stroke

Acute cerebral ischemia (or ischemic stroke) is one of the leading causes of mortality and long-term disability worldwide [83] and it shares with a wide range of neurological disorders the common pathological hallmark of glutamate-mediated excitotoxicity. Ischemic stroke occurs when cerebral blood flow (CBF) is severely reduced, leading to insufficient oxygen and glucose delivery and, ultimately, cell death [84]. Physiological CBF is approximately 50 mL/100 g of brain tissue each minute, and ischemic injury arises when flow drops below 40% of baseline levels, with severity depending on the duration and extent of the interruption [85]. Ischemia can be classified as focal, due to localized vessel occlusion (e.g., embolism or thrombosis), or global, usually following cardiac arrest and systemic hypoperfusion [86].

The focal ischemic lesion evolves through a characteristic spatial and temporal progression. At the lesion center lies the infarction core, where ATP depletion, ionic imbalance, acidosis, and massive glutamate accumulation rapidly trigger necrotic cell death. The surrounding lesion area is the so-called penumbra, a region of partially preserved metabolism due to residual perfusion. Although initially viable, the penumbra is susceptible to delayed apoptotic cell death, particularly due to recurrent peri-infarct depolarizations (PID) or cortical spreading depression ([83, 87]; see also below).

From a temporal perspective, stroke progression can be divided into three main phases. The acute phase begins immediately after CBF reduction, initiating a cascade of molecular events—energy failure, ionic dysregulation, and excitotoxicity. Neurons are particularly vulnerable, with injury observed as early as 30 min after oxygen-glucose deprivation (OGD) [88]. The subacute phase (24–72 h post-stroke) is marked by blood–brain barrier (BBB) disruption and vasogenic edema [89]. The chronic phase, occurring days to weeks later, involves secondary tissue damage with oxidative stress and neuroinflammation [90].

Central to the pathogenesis of ischemic injury is energy failure, which ignites the pathways leading to excitotoxicity. ATP depletion disrupts mitochondrial oxidative phosphorylation, impairing the function of ion pumps such as Na^+^/K^+^-ATPase [83]. This leads to intracellular accumulation of Na^+^ and Cl^−^, K^+^ efflux, and membrane depolarization [91, 92, 93]. Depolarization triggers activation of voltage-gated Ca^2+^ channels (e.g., L-type), resulting in intracellular Ca^2+^ overload [94], which further drives presynaptic glutamate release and establishes a vicious cycle of calcium-dependent excitotoxicity [95]. Multiple pathways contribute to this intracellular calcium accumulation, including NMDA receptor activation, voltage-gated calcium channels, and the Na^+^–Ca^2+^ exchanger [51, 96]. Elevated Ca^2+^ levels promote the pathological release of glutamate, both from presynaptic terminals and through reversed operation of EAATs. Exceeding glutamate then overstimulates ionotropic glutamate receptors—particularly GluN2B-containing NMDARs—which are known to mediate neuronal damage [97, 98].

Astrocytes are profoundly affected during ischemia as well. Under healthy conditions, astrocytic processes envelop synapses and remove approximately 90% of released glutamate via high-affinity EAATs [99, 100], but during ischemia by the reversed operation of these glutamate carriers, glutamate is released into the extracellular space [101].

As a result, astrocytic dysfunction amplifies excitotoxicity, allowing glutamate to persist intra-synaptically, but also causing neurotransmitter leakage outside the synaptic cleft, thus inducing glutamate receptors activation at extra-synaptic sites [102]. These prolong NMDAR activation and promote sustained neurotoxicity. Peri-infarct depolarizations propagate glutamate release from both neurons and glia into adjacent tissue, driving cytotoxic edema, terminal depolarization, and eventual expansion of the infarct core into the penumbra [103].

## 5. Excitotoxicity in Chronic Neurodegenerative Diseases: Some Examples

Glutamate excitotoxicity has been increasingly involved in progressive neuronal loss observed in a range of chronic neurodegenerative diseases, profoundly influencing cognitive functions, such as AD, PD, and Huntington’s disease (HD), to mention some. While each of these CNS disorders is characterized by distinct etiology and CNS-region susceptibility, all share as common thread impaired glutamate homeostasis, elevated intracellular calcium, mitochondrial dysfunction, oxidative stress, and activation of signaling pathways leading to apoptosis [104].

In AD, β-amyloid (Aβ) aggregates and tau pathology are central to disease progression [105], but accumulating evidence suggests that these factors also potentiate excitotoxic signaling. Aβ oligomers have been shown to enhance NMDA receptor activity [106], leading to neuronal oxidative stress [107]. In agreement to the pivotal role of NMDA receptor overactivity in this disorder, memantine, a low-affinity blocker of this receptor, has been used for decades in the treatment of moderate to severe AD [108].

Moreover, astrocytic and neuronal glutamate transporters (e.g., EAAT1/2) are often downregulated or functionally impaired in AD, reducing extracellular glutamate clearance and fostering synaptic toxicity [12].

In PD, excitotoxicity is implicated in the selective degeneration of dopaminergic neurons in the substantia nigra pars compacta (SNpc). These neurons are particularly vulnerable due to their high metabolic demand, pace-making activity, and low calcium-buffering capacity [109]. Mutations in genes such as PARK2 (encoding Parkin) have been shown to destabilize excitatory synapses, increasing susceptibility to glutamate-mediated injury [110]. Furthermore, calbindin-negative SNpc neurons exhibit greater vulnerability to degeneration, highlighting a critical role for intracellular calcium handling in disease progression [111].

In HD, a CAG trinucleotide expansion in the HTT gene results in mutant huntingtin (mHTT), which disrupts synaptic integrity and enhances excitotoxic susceptibility [112]. Striatal medium spiny neurons, particularly GABAergic projections, are selectively degenerated in HD. mHTT has been shown to interact with PSD95 and NMDAR subunits, promoting prolonged receptor activation and calcium overload [113]. Experimental models involving intra-striatal injection of excitotoxins such as kainic acid recapitulate key features of HD pathology, further implicating glutamate receptor overactivation in the neurodegenerative process [114, 115].

Collectively, these findings highlight the role of excitotoxicity as a convergent mechanism of neuronal damage across acute brain injuries and neurodegenerative pathologies. While disease-specific etiologies determine the affected neuronal tissue, the pathological cascade where mitochondrial impairment, oxidative stress, and calcium toxicity are initiated by glutamate dysregulation, is shared and represents a critical therapeutic target. Modulating glutamate homeostasis may thus hold translational potential for slowing excitotoxicity-based disease progression.

With these premises, in this focused review, we briefly introduce the exploitation of graphene-based materials (GBMs) as innovative tools to counteract glutamate release and excitotoxicity. GBMs are an emerging class of nanomaterials recently proposed for new technologies and promising applications in neuroscience.

## 6. New Therapeutic Approaches and Graphene-Based Materials in the CNS

GBMs have garnered a significant role as a component of neuroscience devices due to their outstanding physicochemical properties combined to dimensional and chemical compatibility with biomolecules. Graphene, a single layer of sp^2^-hybridized carbon atoms arranged in a hexagonal lattice, owns its versatile use and developments to the exceptional mechanical strength, high electrical and thermal conductivity, chemical tunability, and a large surface area [116, 117]. These features boost GBMs suitability for a range of biomedical applications [118, 119], especially in the nervous system where electrical signaling, structural support, and specific pharmacological targeting are crucial [120, 121, 122].

**Figure 2:**
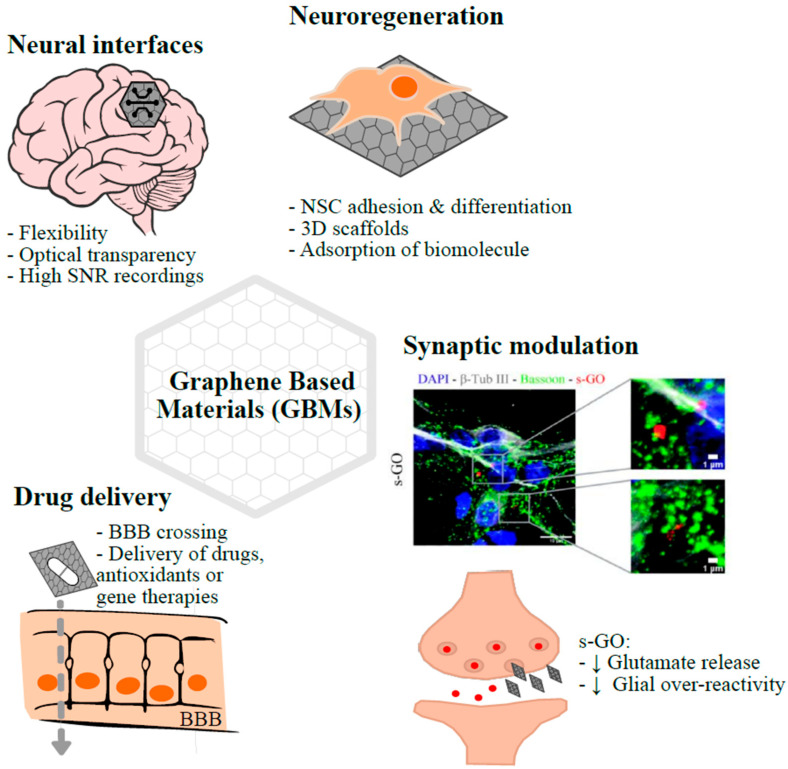
Graphene-based materials for the treatment of excitotoxicity-related neuro-diseases. GBMs have emerged as versatile tools in neuroscience, with potential applications ranging among neural interfacing, neuroregeneration, drug delivery, and synaptic modulation, which may be employed to treat excitotoxicity-related disorders. At the site of excitotoxic lesion, neuronal activity could be monitored through GBMs’ interfaces. The chemo-physical properties of the materials (i.e., flexibility, optical transparency, and good electrical conductivity) could be used to obtain high signal-to-noise ratio recordings, combined with optical imaging of the nervous tissue and poor tissue reactivity to the implant. In GBM-based 3D scaffolds, which can be additionally embedded with growth factors, GBMs can promote the growth and differentiation of neural stem cells and support neuronal synaptic connectivity to repair nervous tissue damaged by the excitotoxic insult. By crossing the BBB, GMBs can serve as a platform for the targeted delivery of drugs, antioxidants, or gene therapies, improving therapeutic efficacy of these compounds. Among GBMs, s-GO, in their pristine form, has been shown to modulate synaptic activity (via reduction in presynaptic glutamate release) and hamper neuroinflammation spreading (by interfering with glial connexin HCs). Such properties can be used to interrupt the glutamate feedback loop in an excitotoxic environment. In the synaptic modulation panel, representative confocal reconstructions of hippocampal neurons treated with s-GO are reported. Cultures were stained for DAPI (in blue) to visualize nuclei, for β-tubulin III (in gray) to visualize neurons, and for bassoon (in green) to identify presynaptic terminals. s-GO (in red) was visualized by confocal reflection mode. The areas in the dashed rectangles in the left image are magnified on the right to show the colocalization of s-GO with presynaptic terminals. From [124], license 6142960988652.

Derivatives of graphene, such as graphene oxide (GO) and reduced graphene oxide (rGO), add further versatility, particularly through their surface functional groups that enhance water solubility and biocompatibility. These materials have been explored across multiple domains of neuroscience, including neural interfaces, scaffolds for nervous tissue regeneration, biosensing, drug delivery, and neural modeling [123] (Figure 2).

### 6.1. Neural Interfaces

One of the more extensively studied applications of GBMs in neuroscience is that of neural interfaces for CNS recording and stimulation. Traditional electrodes, often made of metals like platinum or iridium, suffer from stiffness, degradation, and poor biocompatibility over time [125]. In contrast, graphene’s mechanical flexibility allows them to conform closely to the soft tissue of the brain, minimizing mechanical mismatch and the associated inflammatory responses [126].

Graphene-based microelectrode arrays (MEAs) have demonstrated high signal-to-noise ratios due to graphene’s excellent electrical properties. Moreover, the optical transparency of monolayer graphene enables simultaneous optical imaging and electrical recording, offering new possibilities for neuroimaging applications [127].

Indeed, manufacturing flexible, micro-transistor arrays based on graphene has enabled in vivo high-density mapping of cortical activity in rodents and, in experimental pathological models, has allowed us to measure the propagation of induced spreading depression, a wave of membrane depolarization, with profound metabolic and hemodynamic effects, which plays a central role in diseases such as migraine or ischemic stroke and traumatic brain injury [128]. The stable, high-density recordings provided unprecedented analysis details of the wave dynamics, with the potential to disclose mechanistic insights into disease pathology. Such a device could be exploited as pharmacological screening platform in several distinct neurological disorders. In the context of excitotoxicity, these GBM-based devices could be tested in ischemia to monitor peri-infarct depolarizations (PID) and cortical spreading depression [127] or in neurodegenerative disorders, such as PD, to map and modulate deep brain activity with high precision [129].

### 6.2. Neuroregeneration

GBMs have shown promising results in promoting neuronal growth, differentiation, and synaptic connectivity. For instance, both pristine graphene and GO substrates have been found to support the adhesion and differentiation of neural stem cells into neurons [130] or the connectivity of neurons [131]. This property is attributed to graphene’s ability to adsorb bioactive molecules and provide nanoscale cues that mimic the extracellular matrix [132].

Moreover, graphene scaffolds have been integrated into 3D constructs for CNS injury models, demonstrating functional recovery and enhanced axonal growth [133]. Functionalization of GO with neurotrophic factors or peptides can further enhance its regenerative capacity and targeting efficiency [134]. These approaches hold the potential to promote nervous tissue regeneration at sites damaged by excitotoxicity.

In one of the most recent applications [133], reduced graphene oxide was used to engineer a complex fibrous–porous system, with components mimicking the gray and white matter. The emerging 3D scaffold was tested in vivo in a rat model of spinal cord injury, showing in 4 months both neuroprotective and neuroregenerative abilities, with signs of functional improved recovery.

### 6.3. Drug Delivery

Graphene’s large surface area and π-π stacking ability make it ideal for loading biomolecules, and pharmaceuticals to be delivered to the diseased nervous tissue [135]. In detail, GO, rGO, and graphene quantum dots (GQDs) nanoparticles can be engineered to target CNS oxidative stress and excitotoxic cascades by crossing the blood–brain barrier (BBB), which usually limits drugs’ CNS permeability [136].

GO structure, combining aromatic (sp^2^) and aliphatic (sp^3^) domains, enable the binding of a wide range of therapeutic molecules via non-covalent interactions (e.g., π–π stacking, hydrophobic effects) and covalent functionalization [137, 138]. This makes GO a promising platform for delivering antioxidants, anti-inflammatory agents, gene therapies, and small interfering RNAs (siRNAs) directly to the affected neural tissue.

Functionalization of GO with polymers like polyethylene glycol, chitosan, or dextran enhances biocompatibility and stability in physiological environments, mitigating the risk of aggregation or immune activation [139, 140]. This is particularly crucial given the colloidal nature of GO and its interactions with ions and proteins in biological fluids, which can lead to flocculation or immune responses if not properly stabilized [141].

GO nanocarriers have demonstrated potential to both deliver antioxidant compounds and inhibit oxidative cascades directly. For instance, intranasal delivery of a GO-based nanocomposite carrying dauricine, a compound known to reduce oxidative stress, led to neuroprotective effects in an AD mouse model. GO not only served as a vector but also helped inhibiting amyloid-β aggregation, further mitigating oxidative and inflammatory damage [142, 143].

Similarly, functionalized GO has been used to deliver curcumin—a potent antioxidant and anti-inflammatory compound—alongside other drugs, thus improving therapeutic efficacy while minimizing oxidative side effects [144].

GO has also been used to deliver nucleic acid-based therapeutics, such as siRNA or plasmids targeting redox-sensitive genes. For example, polyethylenimine-functionalized GO was employed to co-deliver doxorubicin (DOX) and Bcl-2-targeted siRNA, demonstrating the dual ability to induce apoptosis in cancer cells while modulating gene expression [145]. Such strategies could be adapted for preventing an excitotoxicity vicious cycle by delivering gene therapies aimed at EAAT upregulation, or mitochondrial function restoration, addressing both oxidative stress and excitotoxicity at the transcriptional level.

In addition, GO functionalization with molecules binding specific receptors can be exploited for targeted drug delivery, i.e., release of a drug at tissue/cells expressing those receptors, improving therapeutic efficacy and reducing side effects. GO functionalized with Fe_3_O_4_ and transferrin enabled DOX delivery across the BBB and into tumor cells, exploiting receptor overexpression and maintaining a pH-dependent release profile to ensure drug activation in acidic tumor microenvironments [146]. This system could be adapted to deliver neuroprotective compounds into excitotoxic microenvironments, such as those present during ischemic injury.

### 6.4. Glutamate Release Modulation via GO Efficiently Limits Excitotoxicity

Among emerging GBM-based strategies aimed at mitigating excitotoxic neuronal injury, small graphene oxide nanosheets (s-GO; lateral size < 500 nm) were shown to possess the ability, when in their pristine form, to directly modulate glutamatergic synaptic transmission [147]. s-GO transiently impaired glutamatergic transmission in the rat hippocampus, both in vitro and in vivo, directly interfering with the presynaptic glutamate release machinery [124, 147]. Mechanistically, it has been proposed, supported by a series of experimental evidence [124, 147], that s-GO adhesion to the cell membrane [148] may impact the exocytic and endocytic synaptic vesicles’ recycling with a transient downregulation in excitatory neurotransmission due to presynaptic vesicle depletion [124]. The synapse specificity and kinetics of s-GO was also demonstrated in other mammalian CNS areas [149] and in the zebrafish in vivo [150]. In addition, recent work showed that the nanomaterial acted by reducing the probability of glutamate release from the presynaptic terminals [151]. s-GO was exploited to transiently reduce glutamatergic activity in basolateral amygdala nuclei in vivo to prevent aversive memory reinforcement and the emergence of anxiety-related behavior [152]. s-GO selectivity for glutamatergic synapses has been linked to the ultrastructural features of these synapses, in respect to inhibitory ones [124, 147]. This property has suggested s-GOs therapeutic potential for disorders characterized by dysregulated glutamatergic neurotransmission.

In vivo, stereotactic injection of s-GO into the dentate gyrus of juvenile rats led to a transient but selective reduction in glutamatergic synaptic activity, which returned to baseline within 72 h post-injection, matching s-GO disappearance from the CNS (shown by confocal Raman maps), suggesting reversible neuromodulatory effects [124]. Similarly, intraspinal administration in zebrafish impaired locomotion due to reduced excitatory signaling in the spinal synaptic network [150]. In a rat behavioral model of post-traumatic stress disorder, characterized by an aberrant synaptic potentiation of glutamatergic synapses in the amygdala, s-GO injected into this brain region during memory consolidation reversed the altered plasticity of excitatory synapses and related pathological, anxiety-related behaviors [151, 152]. Altogether, these results support s-GO ability to normalize glutamatergic synaptic transmission, also in pathological conditions.

s-GO also presents neuroprotective and anti-inflammatory effects targeting glial cells during neuroinflammation. In a mouse model of experimental autoimmune encephalomyelitis (EAE), s-GO injected into the caudal vein, after reaching the spinal cord, reduced astrocyte reactivity and neuronal loss. Such effect was due to the ability of the nanomaterial in interfering with the activity of glial connexin hemichannels, overactivated by neuroinflammation [153].

Recent studies have extended this potential to excitotoxicity-driven pathologies. For instance, Tortella et al. [154] employed an in vitro model of ischemic stroke, using OGD to ignite secondary glutamate-mediated damage in primary hippocampal cultures. s-GO, applied therapeutically 24 h post-insult, significantly reduced neuronal necrosis and preserved neuronal survival, with efficacy comparable to ionotropic glutamate receptor antagonists. In a complementary model of primary excitotoxicity induced by direct exogenous glutamate application, s-GO co-treatment attenuated neuronal death, preserved mitochondrial integrity, and maintained neurite morphology. Electrophysiological recordings further supported the hypothesis that s-GO exerts its neuroprotective effects by downregulating glutamate release and thereby dampening synaptic overactivation. Moreover, s-GO reversed glutamate-induced glial reactivity, suggesting an additional anti-inflammatory mechanism, in agreement with [153]. These findings highlight s-GO as a promising nanomaterial-based intervention to prevent excitotoxic spreading by directly modulating glutamatergic signaling, warranting further exploration in preclinical models of stroke and neurodegeneration.

It is relevant to note that the physical–chemical features of the GMB used in biological applications, together with their concentrations and purity grade, in addition to bio-tolerability and clearance, dictate their specificity when modulating genuine biological functions [155].

## 7. Conclusions

In recent years, our understanding of excitotoxicity has expanded considerably, revealing its central role in a wide range of acute and chronic neurological conditions. Despite advances in identifying the underlying molecular pathways, translating this knowledge into effective clinical treatments has proven challenging, largely due to the difficulty in selectively targeting pathological glutamate signaling without interfering with normal synaptic function. Graphene-based materials, particularly s-GO nanosheets, offer a novel and intriguing direction. Their unique physical and chemical properties, combined with their compatibility with neural tissue and synaptic structures, open the possibility for finely tuned modulation of neuronal activity.

Standardization in synthesis, functionalization, and characterization protocols is essential to ensure reproducibility and biosafety of GBMs for biological applications. Future research should also focus on integrating GBMs with other biomaterials and exploring their synergy with advanced technologies like CRISPR, nanophotonics, and machine learning for responsive neurointerfaces and personalized neurotherapies aimed to maintain cellular homeostasis under excitotoxic conditions.

## Data Availability

Not applicable.

## Abbreviations

The following abbreviations are used in this manuscript:

AD	Alzheimer’s disease
AIF	Apoptosis-inducing factor
AMPA	α-amino-3-hydroxy-5-methyl-4-isoxazolepropionic acid
AMPARs	AMPA receptors
BBB	Blood–brain barrier
CBF	Cerebral blood flow
CBP	CREB-binding protein
CNS	Central nervous system
DOX	Doxorubicin
EAAT/EAATs	Excitatory amino acid transporter(s)
EAE	Experimental autoimmune encephalomyelitis
GAPDH	Glyceraldehyde-3-phosphate dehydrogenase
GBMs	Graphene-based materials
GO	Graphene oxide
GQDs	Graphene quantum dots
HCs	Hemichannels
HD	Huntington’s disease
IP3	Inositol triphosphate
KA	Kainic acid
KARs	Kainate receptors
MEAs	Microelectrode arrays
mHTT	Mutant huntingtin
NCE	Na^+^/Ca^2+^ exchanger
NMDA	*N*-methyl-d-aspartate
NMDAR/NMDARs	NMDA receptor(s)
NO	Nitric oxide
NOS	Nitric oxide synthase
OGD	Oxygen–glucose deprivation
ONOO^−^	Peroxynitrite
PAR	Poly(ADP-ribose)
PARP	Poly(ADP-ribose) polymerase
PD	Parkinson’s disease
PID	Peri-infarct depolarization
PLC	Phospholipase C
PSD/PSD95	Postsynaptic density/Postsynaptic density protein 95
ROS	Reactive oxygen species
SNpc	Substantia nigra pars compacta
VGCCs	Voltage-gated calcium channels
VGSCs	Voltage-gated sodium channels
